# Efficacy of intravenous thrombolysis combined with mechanical stent interventional thrombectomy on acute ischemic stroke

**DOI:** 10.5937/jomb0-35652

**Published:** 2022-10-15

**Authors:** Jun Huang, Ming Zhang, Qingbin Nie, Xinye Zhang, Xin He, Yufeng Yang, Gengsheng Mao

**Affiliations:** 1 The Third Medical Centre Chinese PLA (People's Liberation Army) General Hospital, Department of Neurosurgery, Beijing, China

**Keywords:** thrombolysis, mechanical thrombectomy, acute ischemic stroke, efficacy, tromboliza, mehanička trombektomija, akutni ishemijski moždani udar, efikasnost

## Abstract

**Background:**

To investigate the efficacy and safety of intravenous thrombolysis combined with mechanical stent interventional thrombectomy in the treatment of acute ischemic stroke.

**Methods:**

A retrospective analysis was carried out for clinical data of 118 patients with acute ischemic stroke. The patients enrolled were divided into control group (recombinant tissue plasminogen activator (rt-PA) intravenous thrombolysis) and thrombectomy group (mechanical stent interventional thrombectomy based on rt-PA intravenous thrombolysis). The vascular recanalization rate and clinical efficacy after treatment were compared between the two groups. National Institutes of Health Stroke Scale (NIHSS) was used to identify the degree of neurological impairment in all patients before and after treatment, and Barthel Index was used to assess their activity of daily living. Moreover, the changes in the levels of T-lymphocyte subpopulation in peripheral blood and immuno-inflammatory factors before and after treatment were compared, and prognosis of patients and incidence of adverse reactions were recorded.

**Results:**

The response rate inthrombectomy group (93.2%) was significantly better than that in control group (76.3%). The NIHSS sore and modified Rankin scale (mRS) score after treatment were significantly lower than those before treatment, while the Barthel Index after treatment was distinctly higher than that before treatment. The NIHSS score and mRS score in thrombectomy group obviously declined compared with those in control group at 1 month after treatment. The Barthel Index in thrombectomy group was obviously higher than that in control group at 1 month and 2 months after treatment. Levels of cluster of differentiation 3 (CD3)+, CD3+CD4+, CD4+/CD8+ and natural killer (NK) cells in peripheral blood at 6 months after treatment evidently rose compared with those before treatment, while level of CD3+CD8+ evidently declined compared with that before treatment. In thrombectomy group, levels of CD3+, CD3+CD4+, CD4+/CD8+ and NK cells were markedly higher than those in control group, while the level of CD3+CD8+ was markedly lower than that in control group. Besides, in thrombectomy group, levels of serum osteopontin (OPN), malondialdehyde (MDA) and N-terminal pro-B-type natriuretic peptide (NT-proBNP) were evidently lower than those in control group at 1 month after treatment, while the level of serum superoxide dismutase (SOD) was evidently higher than that in control group. Compared with that in control group, the acute vascular reocclusion rate in thrombectomy group was significantly decreased at 3 months after treatment (10.2% vs. 22.0%).

**Conclusions:**

Intravenous thrombolysis combined with mechanical stent interventional thrombectomy can effectively promote the vascular recanalization, improve the neurological function and activity of daily living of patients, reinforce the immunological function, inhibit the oxidative stress response and improve the prognosis of patients.

## Abstract (sr)

**Uvod:**

Cilj je bio da se istrži efikasnost i bezbednost intravenske trombolize u kombinaciji sa mehanikom intervencijskom trombektomijom stentom u lečenju akutnog ishemijskog moždanog udara.

**Metode:**

Urađena je retrospektivna analiza kliničkih podataka 118 pacijenata sa akutnim ishemijskim moždanim udarom. Uključeni pacijenti su podeljeni u kontrolnu grupu (rekombinantni tkivni aktivator plazminogena (rt-PA) intravenska tromboliza) i grupu za trombektomiju (interventna trombektomija mehaničkim stentom na bazi rt-PA intravenske trombolize). Stopa vaskularne rekanalizacije i klinika efikasnost nakon tretmana upoređivani su između dve grupe. Skala Nacionalnog instituta za zdravlje moždanog udara (NIHSS) je korišćena za identifikaciju stepena neurološkog oštećenja kod svih pacijenata pre i posle lečenja, a Bartel indeks je korišćen za procenu njihove aktivnosti u svakodnevnom životu. Pored toga, upoređene su promene nivoa subpopulacije T-limfocita u perifernoj krvi i imunoinflamatornih faktora pre i posle tretmana i zabeležena je prognoza pacijenata i incidencija neželjenih reakcija.

**Rezultati:**

Stopa odgovora grupe intrombektomije (93,2%) bila je značajno bolja od one u kontrolnoj grupi (76,3%). Rezultati NIHSS bolova i modifikovane Rankinove skale (mRS) nakon tretmana bili su značajno niži od onih pre tretmana, dok je Barthelov indeks nakon tretmana bio znatno viši od onog pre tretmana. NIHSS skor i mRS skor u grupi sa trombektomijom očigledno su opali u poređenju sa onima u kontrolnoj grupi 1 mesec nakon tretmana. Barthelov indeks u grupi sa trombektomijom bio je očigledno viši od onog u kontrolnoj grupi 1 mesec i 2 meseca nakon tretmana. Nivoi klastera diferencijacije 3 (CD3)+, CD3+CD4+, CD4+/CD8+ ćelija prirodnog ubice (NK) u perifernoj krvi 6 meseci nakon tretmana su očigledno porasli u poređenju sa onima pre tretmana, dok je nivo CD3+CD8+ očigledno opao u poređenju sa tim pre lečenja. U grupi sa trombektomijom, nivoi CD3+, CD3+CD4+, CD4+/CD8+ i NK ćelija su bili značajno viši od onih u kontrolnoj grupi, dok je nivo CD3+CD8+ bio značajno niži nego u kontrolnoj grupi. Osim toga, u grupi sa trombektomijom, nivoi serumskog osteopontina (OPN), malondialdehida (MDA) i N-terminalnog pro-B-tipa natriuretičkog peptida (NT-proBNP) bili su evidentno niži od onih u kontrolnoj grupi 1 mesec nakon tretmana, dok je nivo serumske superoksid dismutaze (SOD) bio je evidentno viši nego u kontrolnoj grupi. U poređenju sa onim u kontrolnoj grupi, stopa akutne vaskularne reokluzije u grupi sa trombektomijom je značajno smanjena 3 meseca nakon tretmana (10,2% naspram 22,0%).

**Zaključak:**

Intravenska tromboliza u kombinaciji sa mehaničkom intervencijskom trombektomijom stentom mže efikasno promovisati vaskularnu rekanalizaciju, poboljšati neurološku funkciju i aktivnost svakodnevnog života pacijenata, ojačati imunološku funkciju, inhibirati odgovor na oksidativni stres i poboljšati prognozu pacijenata.

## Introduction

Acute ischemic stroke refers to cerebral infarction caused by cerebral artery occlusion, characterized by high incidence, disability and fatality rates, and its incidence rate is particularly higher in middleage and elderly patients [1] [2]. At present, clinical therapies for acute ischemic stroke focus on vascular recanalization and improvement of blood flow in infarction region, generally represented by intravenous thrombolysis, mechanical thrombectomy, *etc*. [3]. First-line thrombolytic drugs commonly used for clinical treatment are urokinase, and its ways of thrombolysis include intravenous thrombolysisand intra-arterial thrombolysis [4]. However, intravenous thrombolysis can be applied clinically in only part of patients due to certain limitation of therapeutic time window [5]. As interventional therapy progresses recently, mechanical thrombectomy has been increasingly applied, because it has a lower risk of hemorrhage, and can canalize blood vessels within a short time, effectively rescue ischemic penumbra and help shrink the infarct area [6] [7]. Studies indicate that intravascular thrombolysis combined with mechanical thrombectomy contributes to improving the clinical efficacy and relieving neurological impairment in the treatment of acute ischemic stroke [8] [9].

The present study aims to investigate the efficacy and safety of intravenous thrombolysis combined with mechanical stent interventional thrombectomy in the treatment of acute ischemic stroke, and its influence on neurological function, immunological function and prognosis of patients, so as to provide great evidence for the treatment of such patients.

## Materials and methods

### Objects of study

A total of 118 patients with acute ischemic stroke treated in our hospital from January 2016 to March 2021 were selected and divided into control group (n=59) and thrombectomy group (n=59) according to different treatment methods. Recombinant tissue plasminogen activator (rt-PA) intravenous thrombolytic therapy was performed based on routine symptomatic treatment in control group, while mechanical stent interventional thrombectomy was adopted based on rt-PA intravenous thrombolytic therapy in thrombectomy group. Inclusion criteria: 1) patients diagnosed with initial-onset acute ischemic stroke according to skull CT or MRI, and 2) those with time of onset <6 h. Exclusion criteria: 1) patients complicated with severe cardiac, hepatic or renal dysfunction, or accompanied by obvious mental disorders, 2) those with a history of major surgery or trauma, 3) those with severe coagulation disorders or bleeding tendency, or 4) those who used to definitely have neurological or mental disorders. Among the 118 patients, there were 72 males and 46 females aged 37–79 years old, with an average of (59.65±9.59) years old. There were no statistically significant differences regarding the baseline data between the two groups (Table 1, P>0.05). All patients enrolled signed the informed consentin line with the *Declaration of Helsinki*. This study was approved by the Ethics Committee of The Third Medical Centre Chinese PLA (People’s Liberation Army) General Hospital.

**Table 1:** Baseline characteristics of the studied patients Notes: NIHSS: National Institutes of Health stroke scale

Parameters	Thrombectomy group<br> (n=59)	Control group<br> (n=59)	P-value
Age (years)	58.89±9.3	60.24±9.7	0.442
Gender (Male/ Female)	39/20	33/26	0.345
Course of the disease (h)	3.63±0.88	3.58±0.91	0.662
Smoking history (n, %)	19 (32.2%)	23 (39.0%)	0.442
Volume of cerebral infarction (cm^3^)	3.97±1.14	4.06±1.20	0.646
NIHSS score	10.56±3.25	11.49±3.47	0.136
Infarction location (n, %)			0.607
Anterior circulation	37 (62.7%)	34 (57.6%)	
Posterior circulation	22 (37.3%)	25 (42.4%)	
Systemic diseases (n, %)			
Hypertension	29 (49.2%)	32 (54.2%)	0.581
Diabetes Mellitus	14 (23.7%)	11 (18.6%)	0.499
Coronary heart disease	17 (28.8%)	19 (32.2%)	0.689
Atrial fibrillation	9 (15.3%)	7 (11.9%)	0.591

### Treatment methods

In control group, intravenous thrombolytic therapy was performed using rt-PA (Boehringer Ingelheim Pharma GmbH, Germany, registration No. S20110052) at 0.9 mg/kg (the total dose was controlled within 190 mg). 10% of rt-PA was intravenously injected for 1 min first, and then the remaining 90% of rt-PA was added into 100 mL of normal saline and intravenously infused for 1 h.

In thrombectomy group, mechanical thrombectomy was adopted based on the treatment in control group. After local anesthesia with 10 mL of 1% lidocaine, the right femoral artery was punctured using the modified Seldinger technique, a 6F arterial sheath was placed, and heparin (3,000 U, Shanghai No.1 Biochemical & Pharmaceutical Co. Ltd., NMPN H20052319) was added into the kettle. Under the guidance of guide wire, a 6F guiding catheter was inserted and sent to the diseased blood vessel. The location of cerebral artery occlusion and the compensatory status of collateral branch were determined via cerebral angiography. Under the guidance of SilverSpeed-14 micro-guide wire, a Rebar18 micro catheter was inserted through the thrombus, passed over the occluded segment of cerebral artery and reached into the distal branch of the diseased blood vessel. Based on the actual situation of patients, an appropriate Solitaire AB stent was placed for mechanical thrombectomy for no more than 3 times. After the thrombus was removed, the Rebar18 micro catheter was withdrawn, and 30 mL of blood was drawn back from the guiding catheter. Angiography was performed again to confirm whether the flow of the diseased blood vessel is unobstructed. In the case of residual stenosis, angioplasty was performed according to the situation of patients, after which bleeding was stopped using closure devices and the puncture point was bandaged.

At 24 h after treatment, anti-platelet aggregation therapy (oral administration of aspirin 100 mg/d and clopidogrel 75 mg/d) was adopted in both groups for 3 consecutive months. Then the medication was adjusted based on the results of reexamination. The treatment of improving cerebral ischemia (intravenous infusion of 100 mL of butylphthalide and sodium chloride injection, twice/d) and nourishing cranial nerves (intravenous infusion of oxiracetam injection, 4 g/d) was also given for 2 consecutive weeks.

### Observation indexes

The clinical efficacy was assessed at 3 months after operation in the two groups based on the National Institute of Health Stroke Scale (NIHSS) score before and after treatment and clinical symptoms. The definitions were described as following: Cure: After treatment, the NIHSS score declined by >90%, and the clinical symptoms basically disappeared. Markedly effective: After treatment, the NIHSS score declined by 46–89%, the clinical symptoms were significantly alleviated, and the muscle strength of limbson the affected side was significantly increased. Effective: After treatment, the NIHSS score declined by 18–45%, the clinical symptoms were basically controlled, and the muscle strength of limbs on the affected side was increased. Ineffective: After treatment, the NIHSS score declined by <18% or rose, the clinical signs were not improved or the symptoms became worse. The total effective rate of treatment = (cure cases + markedly effective cases + effective cases)/total cases × 100%. After treatment, vascular patency was observed through CT angiography. Vascular recanalization was evaluated using the thrombolysis in cerebral infarction (TICI) grading system, and TICI grade 2 indicates vascular recanalization. Besides, the incidence of adverse reactions during treatment was observed in the two groups, such as intracranial hemorrhage, gastrointestinal hemorrhage, and skin and mucosal ecchymosis.

The patients’ neurological impairment was assessed before and after treatment using the NIHSSscore, and the higher score corresponds to the severer neurological impairment [10]. The patients’ activity of daily living was evaluated before and after treatment using the Barthel index (0–100 points), and the higher score corresponds to the better activity of daily living. Before treatment and at 1 month after treatment, the changes in levels of serum osteopontin (OPN), N-terminal pro-B-type natriuretic peptide (NT-proBNP), superoxide dismutase (SOD) and malondialdehyde (MDA) were detected in the two groups. The levels of serum cluster of differentiation 3 (CD3)^+^, CD4^+^, CD8^+^, CD4^+^/CD8^+^, and natural killer (NK) cells were determined using a FACSCalibur flow cytometer (BD, USA). The patients were followed up, and the residual stenosis rate, 24h symptomatic intracranial hemorrhage rate and survival status were recorded.

### Statistical analysis

Statistical Product and Service Solutions (SPSS) 22.0 software (IBM, Armonk, NY, USA) was used for statistical analysis. Measurement data were expressed as mean±standard deviation (x̅±s), and *t*-test was performed for intergroup comparison and intragroup paired data. Enumeration data were expressed as rate (%), and χ^2^ test or Fisher exact probability test was performed for comparison. P<0.05 suggested the statistically significant difference.

## Results

### Efficacy

The clinical efficacy was evaluated at 3 months after treatment. In thrombectomy group, there were 25 (42.4%) cure cases, 15 (25.4%) markedly effective cases, 14 (23.7%) effective cases and 5 (8.5%) ineffective cases, with the effective rate being 93.2% (54/59). In control group, there were 18 (30.5%) cure cases, 14 (23.7%) markedly effective cases, 13 (22.0%) effective cases and 14 (23.7%) ineffective cases, with the effective rate being 76.3% (45/59). It can be seen that the clinical efficacy was greatly higher in thrombectomy group than that in control group (P=0.024). After treatment, gastrointestinal bleeding occurred in 3 cases and 2 cases, and skin and mucosal ecchymosis occurred in 4 cases and 2 cases, respectively, in thrombectomy group and control group, and they were all improved after symptomatic treatment.

### Neurological function score

Before treatment, there were no statistically significant differences in the NIHSS score, Barthel index and modified Rankin scale (mRS) score between the two groups (P>0.05). At 1, 2 and 3 months after treatment, the NIHSS score and mRS score were obviously lower, while the Barthel index was obviously higher than those before treatment (P<0.05). The NIHSS score and mRS score were obviously lower in thrombectomy group than those in control group at 1 month after treatment (P<0.001), however no statistically significant differences regarding these 2 scores between the two groups at 2 and 3 months after treatment (P>0.05). The Barthel index was obviously higher in thrombectomy group than that in control group at 1 and 2 months after treatment (P=0.003, P=0.012), while results at 3 months after treatment showed no statistically significance between the two groups (P>0.05) (Table 2).

**Table 2:** Comparison of Preoperative and Postoperative NIHSS score, Barthel index and mRS score of patients in the two groups Notes: NIHSS: National Institutes of Health stroke scale; mRS: Modified Rankin scale

Parameters	Thrombectomy<br> group (n=59)	Control group<br> (n=59)	P-value
NIHSS score			
Preoperative	10.56±3.25	11.49±3.47	0.136
1 months Postoperative	6.68±1.41	8.31±1.23	0.001
2 months Postoperative	4.88±1.05	5.23±1.10	0.080
3 months Postoperative	3.47±0.81	3.82±0.84	0.137
Barthel index			
Preoperative	64.67±6.18	65.14±6.49	0.688
1 months Postoperative	79.48±8.24	74.90±8.34	0.003
2 months Postoperative	81.65±9.54	77.22±9.19	0.012
3 months Postoperative	82.17±9.82	79.35±9.63	0.118
mRS			
Preoperative	4.35±0.78	4.21±0.89	0.365
1 months Postoperative	2.44±0.81	3.10±0.85	0.001
2 months Postoperative	2.16±0.59	2.41±0.79	0.054
3 months Postoperative	2.08±0.52	2.26±0.61	0.087

### Levels of immune cells in peripheral blood before and after treatment

Before treatment, no statistically significant differences were found in the levels of CD3+, CD3^+^CD4^+^, CD3^+^CD8^+^, CD4^+^/CD8^+^ and NK cells between the two groups (P>0.05). At 6 months after treatment, the levels of CD3^+^, CD3^+^CD4^+^, CD4^+^/CD8^+^ and NK cells were obviously increased (P<0.05), while the level of CD3^+^CD8^+^ was obviously decreased in the two groups compared with those before treatment (P<0.05). After treatment, the levels of CD3^+^, CD3^+^CD4^+^, CD4^+^/CD8^+^ and NK cells were obviously higher, while the level of CD3^+^CD8^+^ was obviously lower in thrombectomy group than those in control group, showing statistically significant differences (P<0.05) (Table 3).

**Table 3:** Comparison of immunological indicators of patients in the two studied groups Notes: NK: Natural Killer

	Thrombectomy group<br> (n=59)	Control group<br> (n=59)	P-value
CD3^+^ T cell (%)			
Pretreatment	54.18±8.35	53.41±9.09	0.651
Posttreatment	66.37±9.58	59.63±9.14	0.001
CD3^+^CD4^+^ T cell (%)			
Pretreatment	30.33±5.11	29.85±5.23	0.615
Posttreatment	41.41±7.47	37.68±7.05	0.006
CD3^+^CD8^+^ T cell (%)			
Pretreatment	31.22±4.96	30.67±5.37	0.564
Posttreatment	21.51±3.78	24.86±3.72	0.001
CD4^+^/CD8^+^ ratio			
Pretreatment	1.05±0.24	1.03±0.25	0.658
Posttreatment	2.04±0.49	1.79±0.36	0.002
NK cell (%)			
Pretreatment	15.53±6.20	14.69±6.64	0.479
Posttreatment	21.42±7.10	18.21±7.27	0.017

### Expression levels of serum inflammatory factors before and after treatment

**Figure 1:**
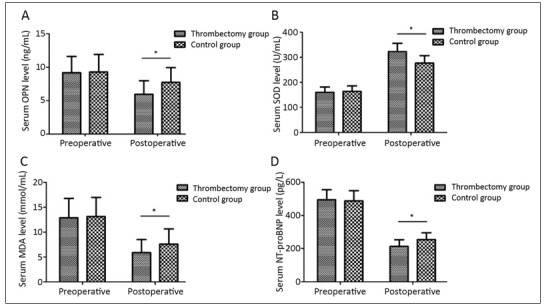
Comparison of pretreatment and posttreatment serum OPN (A), SOD (B), MDA (C), NT-proBNP (D) levels of the studied patients. The difference between preoperative serum OPN (A), SOD (B), MDA (C), NT-proBNP (D) levels of patients in Thrombectomy group and Control group had no statistical significance (P>0.05). Serum OPN (A), MDA (C), NT-proBNP (D) levels of patients were significantly decreased after treatment (P<0.05). Serum SOD (B) level of patients were significantly increased after treatment (P<0.05). Postoperative serum OPN (A), MDA (C), NT-proBNP (D) levels of patients in Thrombectomy group were significantly lower than those of Control group. Postoperative serum SOD (B) level of patients in Thrombectomy group were significantly higher than that of Control group (P>0.05)

The levels of serum OPN, SOD, MDA and NT-proBNP showed no statistically significant differences between the two groups before treatment (P>0.05). At 1 month after treatment, the level of serum OPN declined from (9.17±2.45) ng/mL and (9.29±2.62) ng/mL to (5.95±2.02) ng/mL and (7.74±2.18) ng/mL, the level of SOD rose from (160.36±20.92) U/mL and (164.06±22.19) U/mL to (322.78±33.09) U/mL and (276.81±29.97) U/mL, the level of MDA was decreased from (12.88±3.90) mmol/mL and (13.14±3.83) mmol/mL to (5.87±2.64) mmol/mL and (7.59±3.04) mmol/mL, and the level of NT-proBNP was decreased from (494.38±60.43) pg/L and (487.65±61.72) pg/L to (213.67±39.61) pg/L and (254.68±40.64) pg/L, respectively, in thrombectomy group and control group, displaying statistically significant differences after treatment compared with those before treatment (P<0.05). After treatment, results of the above indexes levels demonstrated statistically significant differences between thrombectomy and control group (P<0.001, P<0.001, P=0.002, P<0.001) (Figure 1).

### Prognosis-related indexes

In thrombectomy group and control group, the proportion of patients in TICIgrade 2b was 64.4% and 57.6%, the residual stenosis rate was 18.6% and 11.9%, the 24 h symptomatic intracranial hemorrhage rate was 6.8% and 10.2%, and the survival rate was 93.2% and 88.1%, respectively, displaying no statistically significant differences (P>0.05). The acute vascular re-occlusion rate at 3 months remarkably declined in thrombectomy group compared with that in control group (10.2% vs. 22.0%, P=0.026) (Table 4).

**Table 4:** Comparison of prognosis indexes of patients in the two groups Notes: TICI: Thrombolysis in cerebral infarction; SICH: Symptomatic intracranial hemorrhage

Parameters	Thrombectomy<br> group (n=59)	Control group<br> (n=59)	P-value
TICI ≥2b	38 (64.4%)	34 (57.6%)	0.450
Residual stenosis	11 (18.6%)	7 (11.9%)	0.306
24 h SICH	4 (6.8%)	6 (10.2%)	0.509
Acute vascular<br> re-occlusion	6 (10.2%)	13 (22.0%)	0.026
Survival rate	55 (93.2%)	52 (88.1%)	0.342

## Discussion

Due to varying degrees of stenosis and occlusion of carotid and vertebral arteries, or insufficiency of cerebral blood supply and atrial fibrillation-induced embolus shedding, acute cerebrovascular occlusion is caused, leading to acute ischemic necrosis of brain tissues, which is known as acute ischemic stroke. The disease is manifested as limb weakness, language disorder, distortion of commissure, gaze, unconsciousness, lethargy and coma, and the symptoms cannot be spontaneously relieved budget progressively worse. Therefore, prompt diagnosis and targeted treatment are of great significance to the improvement of prognosis [11] [12].

Currently, intravenous thrombolysis has become an important clinical treatment means of acute ischemic stroke. It is mainly used in the early rescue of patients with acute ischemic stroke, and its time window lasts for 4.5 h, in which thrombolytic drugs are intravenously injected to dissolve the emboli in the infarction lesion, improve the blood circulation, and reduce the area of infarction lesion. Therefore, it is the most effective method to reduce the disability rate of patients with acute ischemic stroke [13] [14]. In recent years, arterial recanalization technique, namely intra-arterial stent thrombectomy, has been gradually applied in the treatment of acute ischemic stroke, and its time window lasts for 16-24 h, in which the stent is placed in the patient's stenotic artery and then withdrawn after being released for a period of time. As a result, the intra-arterial embolus can be effectively sucked out. Moreover, as mechanical thrombectomy, intra-arterial thrombectomy has higher efficiency, and can not only effectively seize the time window, but also promote the establishment of collateral circulation in stenotic or occluded arteries, thereby improving the blood perfusion of brain tissues and relieving the cerebral ischemic response [15] [16]. A study demonstrated that intravascular thrombolysis combined with mechanical thrombectomy can not only control the condition of disease, but also improve the prognosis and lower the clinical fatality rate in the treatment of acute ischemic stroke [17]. Besides, intravenous thrombolysis combined with mechanical thrombectomy can effectively clear the thrombus in occluded vessels, restore the forward flow in blood vessels, and reduce brain cell damage. In the case of residual stenosis, the Solitaire AB stent can be released for angioplasty [18].

In this study, the total effective rate of clinical treatment in thrombectomy group was significantly higher than that in control group. At 1, 2 and 3 months after treatment, the NIHSS score and mRS score linearly declined in the two groups compared with those before treatment. Both NIHSS score and mRSscore were significantly lower in thrombectomy group than those in control group at 1 month after treatment, and the Barthel index was remarkably higher in thrombectomy group than that in control group at 1 and 2 months after treatment. The acute vascular re-occlusion rate at 3 months after treatment remarkably declined in thrombectomy group compared with that in control group, consistent with the research results of Mueller-Kronast et al. [19]. It can be seen that intravenous thrombolysis combined with mechanical thrombectomy can improve the clinical efficacy, benefit the recovery of neurological function, raise the activity of daily living, and greatly ameliorate the prognosis in the treatment of acute ischemic stroke. Patients with acute ischemic stroke may suffer from immune imbalance (abnormal levels of CD3^+^, CD4^+^ and CD4^+^/CD8^+^), leading to pneumonia, intracranial edema, infection and other complications, and worsening brain injury. In this study, at 6 months after treatment, the levels of CD3^+^, CD4^+^, CD4^+^/CD8^+^ and NK cells were increased, while the level of CD8^+^ was decreased in the two groups compared with those before treatment, and there were statistically significant differences. After treatment, the increases in the levels of CD3^+^, CD4^+^, CD4^+^/CD8^+^ and NK cells, and the decrease in the CD8^+^ level were more obvious in thrombectomy group than those in control group, indicating that intravenous thrombolysis combined with mechanical thrombectomy can help improve the immune function and accelerate the recovery of patients in the treatment of acute ischemic stroke.

In addition, the levels of serum OPN, SOD, MDA and NT-proBNP are closely related to the occurrence and development of acute cerebral infarction and neurological damage. Therefore, the changes in the above indexes can be monitored in clinic to judge the change in patients' condition and therapeutic effect. In this study, at 1 month after treatment, the levels of serum OPN, MDA and NT-proBNP were far lower in thrombectomy group than those in control group, while thrombectomy group had a far higher level of SOD than control group. The above findings are consistent with previous research results [20], suggesting that intravenous thrombolysis combined with mechanical thrombectomy can regulate the expressions of neurological function-related factors and inhibit the body's oxidative stress response, thereby promoting the recovery of neurological function and reducing the neurological impairment.

However, limitations still existed in this retrospective study. The limited sample size, short and incomplete follow-up weakened the evidence level. Therefore, the conclusion in this study needs to be further verified through large-sample multi-center long-term follow-up studies in the future.

## Conclusions

Intravenous thrombolysis combined with mechanical stent interventional thrombectomy can effectively promote the vascular recanalization, improve the patient’s neurological function and activity of daily living, enhance the body’s immune function, inhibit the body’s oxidative stress response, and ameliorate the patient’s prognosis in the treatment of acute ischemic stroke.

## Dodatak

### Conflict of interest statement

All the authors declare that they have no conflict of interest in this work.
